# Functional analysis of the biochemical activity of mammalian phosphatidylinositol 5 phosphate 4-kinase enzymes

**DOI:** 10.1042/BSR20182210

**Published:** 2019-02-19

**Authors:** Swarna Mathre, K. Balasankara Reddy, Visvanathan Ramya, Harini Krishnan, Avishek Ghosh, Padinjat Raghu

**Affiliations:** 1 National Centre for Biological Sciences, TIFR-GKVK Campus, Bellary Road, Bangalore 560065, India; 2 School of Life Sciences, Manipal Academy of Higher Education, Manipal 576104, Karnataka, India

**Keywords:** enzyme activity, kinases, lipid mediators, phosphatidylinositol

## Abstract

Phosphatidylinositol 5 phosphate 4-kinase (PIP4K) are enzymes that catalyse the phosphorylation of phosphatidylinositol 5-phosphate (PI5P) to generate PI(4,5)P_2_. Mammalian genomes contain three genes, *PIP4K2Α, 2B* and *2C* and murine knockouts for these suggested important physiological roles *in vivo*. The proteins encoded by *PIP4K2A, 2B* and *2C* show widely varying specific activities *in vitro*; PIP4K2A is highly active and PIP4K2C 2000-times less active, and the relationship between this biochemical activity and *in vivo* function is unknown. By contrast, the *Drosophila* genome encodes a single PIP4K (dPIP4K) that shows high specific activity *in vitro* and loss of this enzyme results in reduced salivary gland cell size *in vivo*. We find that the kinase activity of dPIP4K is essential for normal salivary gland cell size *in vivo*. Despite their highly divergent specific activity, we find that all three mammalian PIP4K isoforms are able to enhance salivary gland cell size in the *Drosophila* PIP4K null mutant implying a lack of correlation between *in vitro* activity measurements and *in vivo* function. Further, the kinase activity of PIP4K2C, reported to be almost inactive *in vitro*, is required for *in vivo* function. Our findings suggest the existence of unidentified factors that regulate PIP4K enzyme activity *in vivo*.

## Introduction

The phosphorylation of lipids to generate phospho-variants is a strategy used to generate signalling molecules that encode information on ongoing cellular processes. A widespread mechanism of cell signalling in eukaryotes is the phosphorylation of phosphatidylinositol at positions 3, 4 and 5 to generate a set of seven second messengers [1]. The addition of these phosphate groups to the inositol ring is catalysed by phosphoinositide kinases, enzymes with a high degree of substrate specificity both for the substrate they use and the position on the inositol ring at which they catalyse phosphorylation. The generation of the lipid phosphatidylinositol 4,5 bisphosphate [PI(4,5)P_2_] exemplifies these features (reviewed in [2]). PI(4,5)P_2_ can be synthesised by two enzymatic pathways: (i) the phosphorylation of phosphatidylinositol 4-phosphate (PI4P) at position 5 of the inositol head group; the enzymes that catalyse this reaction are phosphatidylinositol 4 phosphate 5-kinase (PIP5K). PI4P and PI(4,5)P_2_ are substantially more abundant in eukaryotic cells and previous studies have suggested that the bulk of the PI(4,5)P_2_ in animal cells is synthesised by this pathway [3] and (ii) the phosphorylation of phosphatidylinositol 5-phosphate (PI5P) at position 4 of the inositol head group; the enzymes that catalyse this reaction are the phosphatidylinositol 5 phosphate 4-kinases (PIP4K). PI5P is found at far lower abundance than PI4P in cells and thus the PI(4,5)P_2_ pool generated by PIP4K is likely to be quantitatively minor; hence, it has been proposed that the biochemical relevance of PIP4K enzymes may be to control the levels of its substrate PI5P (reviewed in [2]). Thus the functional significance of PIP4K enzymes remains a topic of interest.

PIP4K activity was first described in 1997 by the Cantley lab as an enzyme that could catalyse the conversion of PI5P (but not PI4P) into PI(4,5)P_2_ [4]. Subsequent studies have also suggested that these enzymes are able to use PI5P as substrate to generate the product PI(4,5)P_2_ (reviewed in [2]). While PIP5K genes are found in all eukaryotes, genes encoding PIP4K are thus far described in metazoan model genomes but not those of unicellular eukaryotes. While invertebrate models such as *Drosophila* and *Caenorhabditis elegans* contain a single gene encoding for PIP4K, mammalian genomes contain three genes encoding PIP4K isoforms. Each gene appears to underpin key biological functions; loss of PIP4K2A (also called PIP4Kα) and PIP4K2B (also called PIP4Kβ) is able to reduce the rate of tumour growth in p53^−/−^ mice [5]; loss of PIP4K2C (also called PIP4Kγ) triggers an autoimmune response in mouse models [6] and recently PIP4K2C has been shown to control the clearance of protein aggregates in a cellular model of Huntington’s disease [7]. PIP4K enzymes or their substrate PI5P have been proposed to regulate diverse subcellular processes including nuclear function [8], membrane transport [9–11] *m*ammalian *T*arget *o*f *R*apamycin (mTOR) signalling [12] and autophagy [13]. A number of isoforms of PIP4K from multiple species have been cloned, expressed and shown to have conserved biochemical activity [12,14]. Surprisingly, when studied *in vitro*, these polypeptides show a very large range of specific activities. Mammalian PIP4K2A shows high specific activity being 100-fold more active than PIP4K2B and 2000-fold more active compared with PIP4K2C [14]. Remarkably, while the single PIP4K in the *Drosophila* genome shows high specific activity comparable with PIP4K2A, the only PIP4K gene in *C. elegans* (*ppk-2*) shows very low activity similar to that seen for PIP4K2C [14]. The reason for this widely varying activity of PIP4K as measured *in vitro* and its relevance to *in vivo* functions remains unclear.

When expressed and studied *in vitro, Drosophila* PIP4K (*dPIP4K*) has been shown to have high specific activity [12] and a substrate specificity similar to that of PIP4K2A. *In vitro* activity assays have typically been performed (for e.g. [12,15] with a fixed amount of purified, recombinant enzyme incubated with a defined amount of substrate in a reaction buffer in a test tube). A null allele of dPIP4K (*dPIP4K^29^*) shows elevated levels of PI5P the substrate of PIP4K; the elevated PI5P levels can be reversed by reconstituting with a wild-type dPIP4K but not a kinase dead transgene of the enzyme [12]. *dPIP4K^29^* larvae show delayed growth and development; in the larval salivary glands, the average size of cells is reduced and this is associated with a reduction in complex 1 of mTOR (TORC1) activity [12]. These phenotypes can be reversed by pan-larval reconstitution of *dPIP4K^29^* with a wild-type transgene. dPIP4K has also been shown to regulate clathrin-dependent endocytosis of G-protein coupled receptors and the size of the Rab5 compartment in *Drosophila* cells; this phenotype appears independent of the kinase activity of the enzyme [11].

In the present study, we reconstituted PIP4K function in the salivary glands of *dPIP4K^29^*, a loss of function mutant in the single dPIP4K. Such reconstitution with dPIP4K was able to rescue the cell size phenotype and this rescue was dependent on the kinase activity of the enzyme. Further we find that all isoforms of mammalian PIP4K, irrespective of their specific activity as measured *in vitro*, were individually able to rescue the reduced cell size phenotype of *dPIP4K^29^*. These observations suggest that the *in vitro* activity of PIP4K enzymes may not reflect their activity *in vivo*. Thus additional factors in intact cells may regulate the activity of PIP4K enzymes.

## Experimental procedures

### Generation of constructs and flies

Human PIP4K constructs were a generous gift from Dr Jonathan Clarke, University of Cambridge, U.K. The genes were subcloned into the *Drosophila* expression vector pJFRC (Addgene) modified from [pJFRC-MUH from Gerald Rubin (Addgene plasmid # 26213)] to carry a C-terminal eGFP. All three genes were amplified with primers incorporating XhoI in the forward and BamHI in the reverse; these sites were used to subclone these genes in pJFRC-eGFP. Thus generated clones were first tested for expression in S2R+ cells. Once the expression and localisation was confirmed, these constructs were microinjected to generate transgenic *Drosophila* strains. Transgenic flies were generated by site-specific recombination at attP2 sites on either the second or third chromosome.

### Cell size measurement of salivary glands

Uniformly non-crowded vials were reared to obtain wandering third instar larvae. These were isolated and dissected in 1× PBS, fixed in 4% PFA either at 4°C for 20 min or at room temperature for 20 min. Fixed glands were stained with BODIPY-FL-488 for 3 h after which the glands are stained with either TOTO3 for 5 min or DAPI for 10 min at room temperature. The glands are then washed overnight in 1× PBS and mounted in 70% glycerol. Stained glands were imaged either on an Olympus FV1000 or FV3000 confocal microscope at 20× magnification. To obtain an image of the whole gland, multiple frames of confocal stacks were first obtained which were later stitched using the image stitching tool in ImageJ. Whole gland images were analysed for volume measurements using *Volocity* (version 5.5.1, PerkinElmer Inc.). For obtaining average cell size measurements, the whole gland volume was divided by the total number of nuclei present in the gland.

### Sequence analysis

Multiple sequence alignment of dPIP4K and all three human PIP4Ks were obtained using Clustal O [16]. The phylogenetic tree for the above proteins was obtained using neighbour joining method (MEGA6) [17]. Sequence similarity was calculated for individual sequence alignments between dPIP4K and the three human isoforms.

### Imaging of salivary glands for localisation

Salivary glands dissected were fixed in PLP fixative [18] for 20 min at room temperature. Fixed glands were further washed in 0.1% PTX and stained with DAPI for 5 min at room temperature. The salivary glands were imaged for localisation at 40×/60× on an Olympus FV3000 confocal microscope.

### S2R+ cell samples

S2R+ cells were transfected with GFP-tagged constructs using Effectene-based transfection. Transfected cells were cultured for 48 h before harvesting them for either plating in dishes and confocal imaging, or for isolating protein samples for immunoblotting. For imaging, the cells once seeded onto coverslip dishes were left for an hour to settle. Cells were fixed in 2.5% PFA at room temperature for 20 min after which washes were carried out in M1 Buffer (0.15 mM NaCl, 5 mM KCl, 1.32 mM CaCl_2_, 2.13 mM MgCl_2_, ∼20 mM HEPES). Imaging of S2R+ cells was performed on an FV3000 confocal microscope at 60× magnification.

### Western blotting

A minimum of six pairs of salivary glands were isolated from wandering third instar larvae for lysate preparation. Salivary glands were homogenised by bath sonication for 3 min in lysis buffer containing 50 mM Tris/Cl, pH 7.5, 1 mM EDTA, 1 mM EGTA, 1% Triton X-100, 50 mM NaF, 0.27 M Sucrose, 0.1% β-mercaptoethanol and freshly added protease and phosphatase inhibitors (Roche). Following addition of Laemelli buffer, samples were heated at 95°C for 5 min. Following SDS/PAGE, proteins were transferred on to nitrocellulose membrane.

Lysates of S2R+ cells were prepared by first washing the cells in 1× PBS twice. Then cells were lysed in the lysis buffer mentioned previously. Lamelli buffer was added to the sample and heated at 95°C for 5 min.

SDS/PAGE (10% gel) was used to separate proteins followed by wet-transfer onto nitrocellulose membrane. The membrane was blocked with Blotto (in 0.1% Tween 20 in 1× PBS) and primary antibody incubations were done in the same solution overnight. Washes were done in 0.1% Tween in 1× PBS. Secondary antibody incubations were done for 2 h at room temperature after which, the membrane was visualised using ECL reagent (GE Healthcare).

Antibodies used: anti-GFP - GFP(B-2) sc9996 (mouse) from Santa Cruz Biotechnology, anti-tubulin (E7-c), (mouse) DSHB and anti-Actin (Rabbit) A5060 from Sigma. Anti-dPIP4K antibody (Rabbit) was generated in the lab as described previously [12].

### PI5P 4-kinase assay

Lipid substrates (6 μM PI5P) and 20 μM of phosphatidylserine were vacuum dried and resuspended in 10 mM Tris pH 7.4. This preparation was sonicated for 2 min in a bath-sonicator for micelle formation; 50 µl of 2× PIP kinase reaction buffer (100 mM Tris pH 7.4, 20 mM MgCl_2_, 140 mM KCl, and 2 mM EGTA) containing 20 µM ATP, 5 µCi [γ-^32^P] ATP and ∼10 µg total protein from S2R+ cell lysates was added to the micelles and incubated at 30°C for 16 h. Lipids were extracted and resolved by 1D TLC (45:35:8:2 :: chloroform: methanol: water:25% ammonia) which was later imaged using phosphorimager.

## Results

### The kinase activity of dPIP4K regulates salivary gland cell size

**Figure 1:**
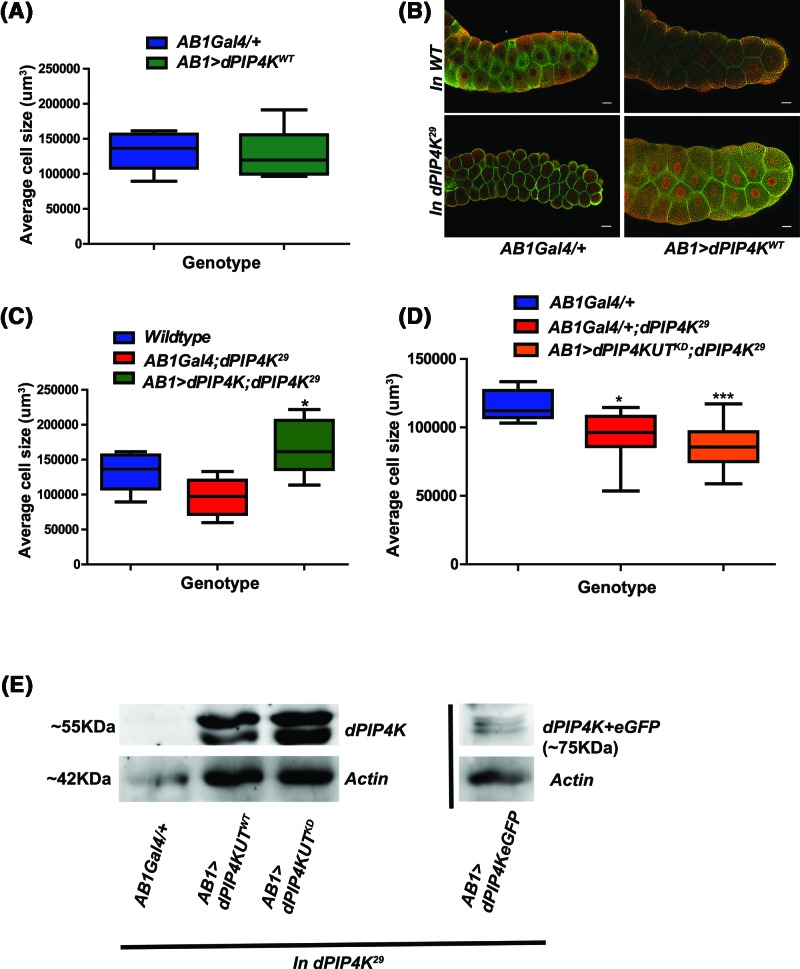
dPIP4K functions in modulating cell size in larval salivary glands (**A**) Graph representing average cell size measurement (μm^3^) as mean ± S.E.M. of salivary glands from wandering third instar larvae of *AB1Gal4/+* and overexpression of *AB1>dPIP4K^WT^, n*=5. No significant change was observed when analysed by unpaired *t* test. (**B**) Representative confocal images of salivary glands from the genotypes a. *AB1Gal4/+* b. *AB1>dPIP4K^WT^* c*. AB1Gal4/+;dPIP4K^29^* and d. *AB1>dPIP4K^WT^;dPIP4K^29^* showing cell size changes quantified in the earlier graph. Cell body is marked green by Bodipy conjugated lipid dye, nucleus is marked by TOTO3 shown in red. Scale bar indicated at 50 μm. Images are contrast adjusted for representation. (**C**) Graph representing average cell size (μm^3^) measurements as mean ± S.E.M. on reconstitution of *dPIP4K^WT^* in the background of *dPIP4K^29^* as compared with *AB1Gal4/+;dPIP4K^29^, n*=8. *P-value <0.05. (**D**) Graph representing average cell size (μm^3^) measurements as mean ± S.E.M. on reconstitution of *dPIP4KUT^KD^* (where ‘UT’ indicates untagged protein) in the background of *dPIP4K^29^* as compared with *AB1Gal4/+; dPIP4K^29^, n*=8. *P-value <0.05, ***P-value <0.01. (**E**) Comparison of protein levels between dPIP4KUT^WT^ and dPIP4KUT^KD^ as compared with *AB1Gal4/+;dPIP4K^29^* control in larval salivary glands, as seen on a Western blot probed by dPIP4K antibody. dPIP4K::eGFP which migrates ∼25 kDa higher than the untagged protein represented separately. Actin was used as the loading control for all.

We tested the ability of dPIP4K to rescue the reduced cell size phenotype in *dPIP4K^29^*. Previous work has shown that pan-larval reconstitution of dPIP4K *(da > dPIP4K^WT^; dPIP4K^29^*) in *dPIP4K^29^* can rescue the reduced size of salivary gland cells [12]. In this study, we selectively expressed dPIP4K in salivary gland cells using a salivary gland-specific GAL4. Overexpression of dPIP4K in wild-type salivary glands did not change cell size (Figure 1A). When dPIP4K was selectively reconstituted in the salivary gland cells of *dPIP4K^29^* (*AB1>dPIP4K^WT^; dPIP4K^29^*) cell size was rescued (*P*-value: 0.0123) (Figure 1B,C). To test the requirement of kinase activity in rescuing cell size, we reconstituted *dPIP4K^29^* salivary glands with a kinase dead version (*AB1>dPIP4K^KD^; dPIP4K^29^*) (D271A) that shows no kinase activity when tested *in vitro*. When *dPIP4K^KD^* was reconstituted in *dPIP4K^29^* salivary glands, it was unable to rescue the reduced cell size (*P*-value: 0.3941) seen in this mutant (Figure 1D) although dPIP4K^KD^ protein levels were equivalent to that of wild-type (Figure 1E).

### Heterologous expression of human PIP4K in *Drosophila* cells

**Figure 2:**
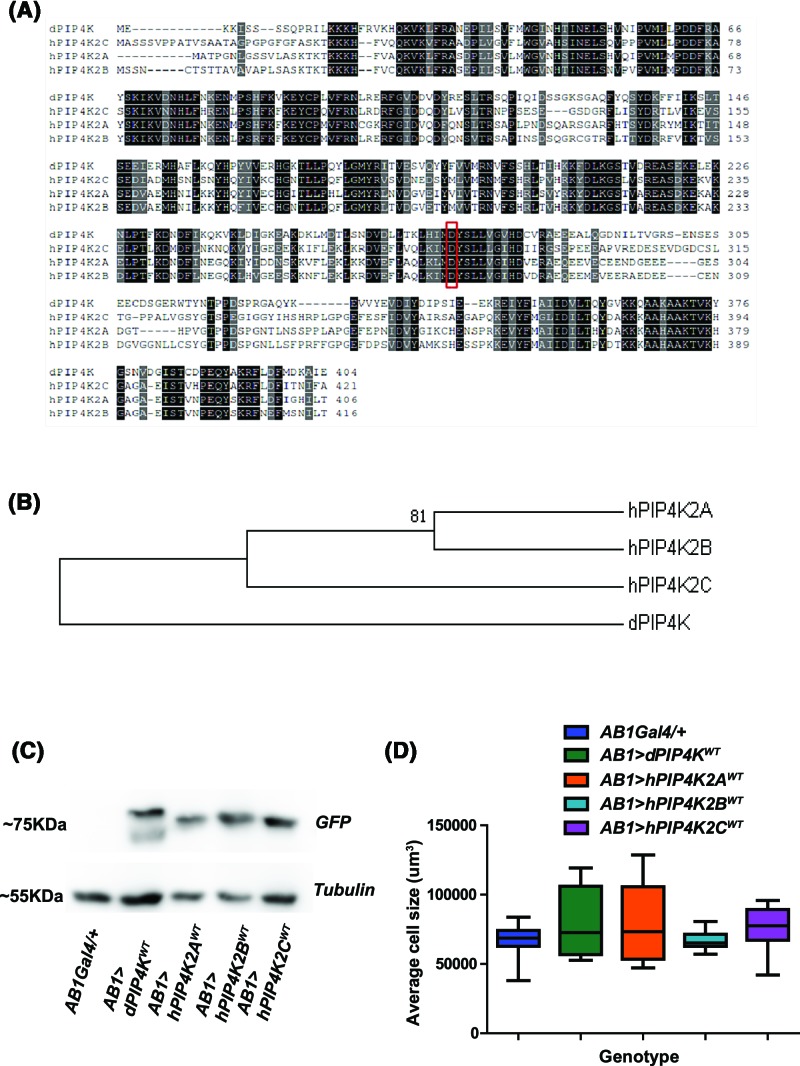
Heterologous expression of mammalian PIP4Ks in *Drosophila* cells and tissue (**A**) Protein alignment of the three human PIP4Ks against dPIP4K protein sequence. The conservation of residues has been marked from 60 to 80% (grey to black). The aspartic acid residue (D in the functional motif ‘MDYSLL’) has been marked with red box. (**B**) Phylogenetic tree representing the evolutionary distance between different PIP4Ks are used in the present study. The neighbour joining mode in MEGA 6 has been used to obtain the phylogenetic tree. The tree is represented at 50% consensus. The node with bootstrap value above 75% has been marked. (**C**) Protein levels in salivary glands of wild-type larvae overexpressing dPIP4K or the three human PIP4Ks as compared with *AB1Gal4/+* probed for GFP tag on Western blot. Tubulin is used as a loading control. (**D**) Graph representing average cell size measurement (μm^3^) as mean ± S.E.M. of salivary glands from wandering third instar larvae expressing *dPIP4K^WT^, hPIP4K2A^WT^, hPIP4K2B ^WT^* and *hPIP4K2C^WT^* in salivary glands as compared with *AB1Gal4/+. n*=5, no significant change was observed when analysed by unpaired *t* test.

The human genome contains three genes (*PIP4K2A, PIP4K2B* and *PIP4K2C*) that encode PIP4K enzymes. *In silico* analysis reveals a broad degree of sequence conservation among these three genes (Figure 2A). hPIP4K2A and hPIP4K2B show more than 50% identity with dPIP4K whereas hPIP4K2C shows close to 50% (47.9%) identity. hPIP4K2B shows highest similarity to dPIP4K in terms of protein sequence (71%), hPIP4K2A shows 67.7% similarity and hPIP4K2C shows least similarity at 62.5%. Collectively, all three proteins show similarity above 60% to dPIP4K. Phylogenetic analysis reveals that the human PIP4K isoforms cluster together and dPIP4K is represented as a separate branch in the evolutionary tree (Figure 2B). We generated constructs to express each of the three human PIP4K genes fused to GFP (PIP4K::GFP) in *Drosophila* S2R+ cells. Each of the three human PIP4K::GFP was expressed as a polypeptide of the predicted *M*_r_ (Supplementary Figure S1B). Immunofluorescence analysis revealed that each human PIP4K::GFP showed a distinctive subcellular distribution when expressed in S2R+ cells that broadly recapitulate observations reported in mammalian cells (Supplementary Figure S1A) [19–21]. While hPIP4K2A::GFP was diffusely distributed (Supplementary Figure S1A), a substantial fraction of hPIP4K2B::GFP was found in the nucleus (Supplementary Figure S1A) and hPIP4K2C::GFP appeared largely distributed in a distinctive intracellular punctate fashion (Supplementary Figure S1A).

We generated transgenic flies to allow expression of these human PIP4K::GFP proteins *in vivo*. When expressed in salivary glands, all three human PIP4K isoforms showed protein expression at equivalent levels and similar to dPIP4K::GFP under the same conditions (Figure 2C). When expressed in salivary gland cells, hPIP4K2A::GFP was localised to the plasma membrane and was also observed in the cytoplasm (Supplementary Figure S1C(d,e,f)); hPIP4K2B::GFP was observed both at the plasma membrane and within the nucleus (Supplementary Figure S1C(g,h,i)) and hPIP4K2C::GFP was observed in punctate intracellular compartments (Supplementary Figure S1C(j,k,l)). Under similar conditions, dPIP4K::GFP showed localisation to the plasma membrane and the cytoplasm but was not detected in the nucleus or punctate intracellular compartments (Supplementary Figure S1C(a,b,c)). When dPIP4K is overexpressed in otherwise wild-type glands, there is no impact on average cell size (Figure 2D). Similar to that seen with dPIP4K, overexpression of each of the human PIP4K::GFP in wild-type salivary glands (*AB1> PIP4K::GFP*) did not result in any significant change in salivary gland cell size (Figure 2D).

### hPIP4K2A and hPIP4K2B can rescue the cell size defect of *dPIP4K^29^*

**Figure 3:**
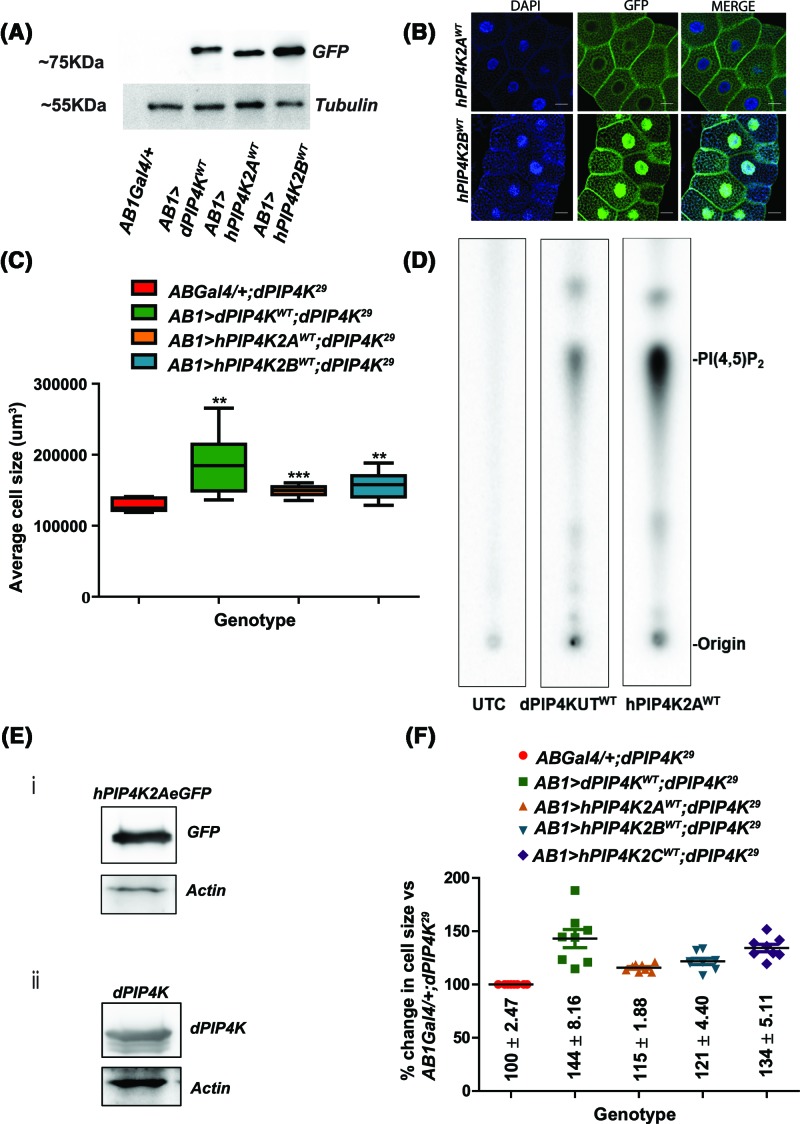
Reconstitution of the high specific activity PIP4Ks:hPIP4K2A and hPIP4K2B reverts cell size reduction seen in *dPIP4K^29^* (**A**) Western blot showing the protein levels of human PIP4Ks as compared with dPIP4K seen using anti-GFP. Tubulin was used as the loading control. (**B**) Confocal images of salivary glands expressing GFP-tagged hPIP4K2A and hPIP4K2B in background of *dPIP4K^29^* along with DAPI staining the nucleus in blue. Scale bar indicated at 20 μm. Images are contrast-adjusted for representation. (**C**) Graph representing average cell size measurement (μm^3^) as mean ± S.E.M. for heterologous reconstitution of hPIP4K2Aand hPIP4K2A as compared with *dPIP4K^WT^* expression in *AB1Gal4* in *dPIP4K^29^, n*=8, **P-value <0.05, ***P<0.001. (**D**) TLC image showing activity for hPIP4K2A and dPIP4KWT as compared with an untransfected control (UTC). The expected spot representing PIP2 generation is marked on the right. (**E**) Western blot image showing protein levels of (i) hPIP4K2A and (ii) dPIP4K^WT^ in the lysate used for kinase assay. Actin was used as a loading control in both cases. (**F**) Ratiometric analysis of change in cell size measured as a % change is represented for reconstitution of *dPIP4K^WT^, hPIP4K2A, hPIP4K2B* and *hPIP4K2C* with respective individual experimental *AB1Gal4/+;dPIP4K^29^* control.

The three human PIP4Ks are known to vary in their specific activity as measured using *in vitro* assays [14]. hPIP4K2A, having highest *in vitro* activity and hPIP4K2B which has approximately 100-fold less activity as compared with hPIP4K2A, yet substantial as measured *in vitro*, were reconstituted individually in *dPIP4K^29^*. When reconstituted in salivary gland cells of *dPIP4K^29^*, we found that both hPIP4K2A and hPIP4K2B were expressed at protein levels similar to dPIP4K (Figure 3A). They were also localised in a manner similar to that reported in mammalian cells with hPIP4K2A largely seen to be cytosolic, along with some plasma membrane localisation, whereas hPIP4K2B was present in nucleus along with cytosolic and membrane localisation (Figure 3B). Under these conditions, hPIP4K2A (*AB1>hPIP4K2A^WT^;dPIP4K^29^*) and hPIP4K2B (*AB1>hPIP4K2B^WT^;dPIP4K^29^*) were both able to significantly increase cell size in *dPIP4K^29^*, although their ability to restore cell size back to wild-type levels was not as great as with *dPIP4K^WT^* (*AB1>dPIP4K^WT^;dPIP4K^29^*) (Figure 3C). Lysates from S2R+ cells transfected with dPIP4K and hPIP4K2A were tested for enzyme activity; under these conditions, both enzymes showed activity in converting PI5P into PI(4,5)P_2_ (Figure 3D,E).

### hPIP4K2C can rescue cell size defects seen in *dPIP4K^29^*

**Figure 4:**
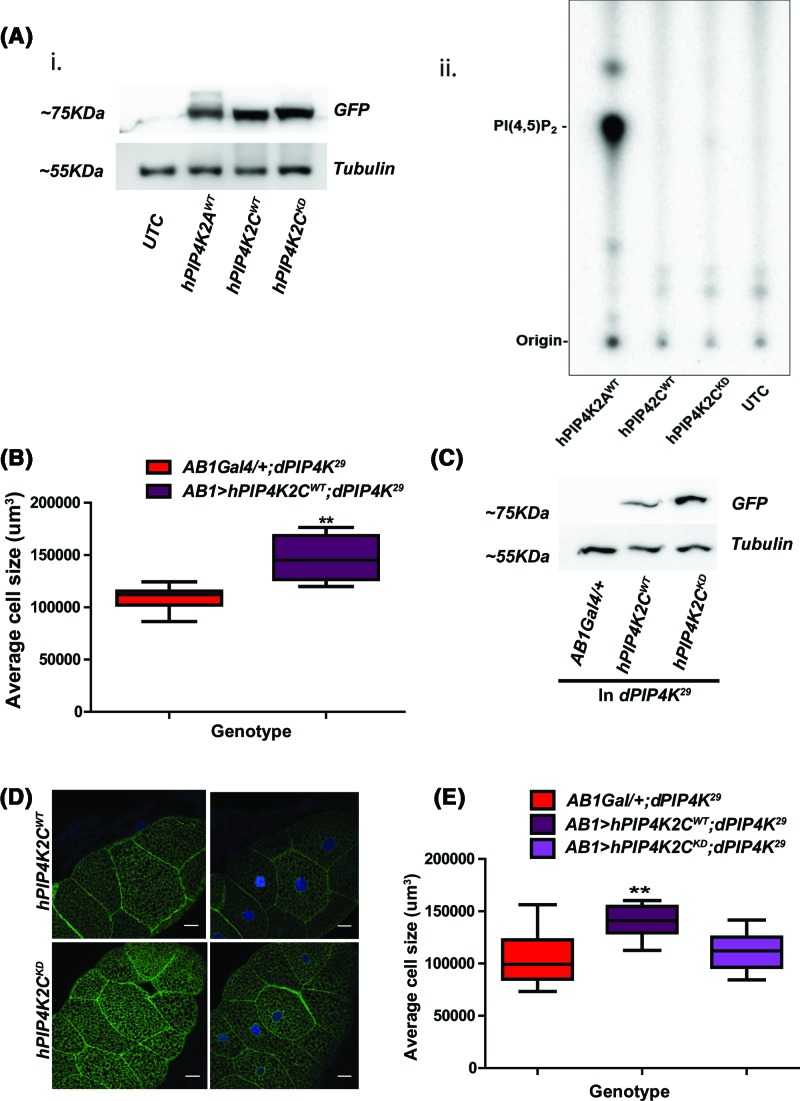
Reconstitution of low specific activity containing PIP4K: *hPIP4K2C* restores the cell size reduction seen in *dPIP4K^29^* (**A**) PI5P kinase activity assay. (i) Immunoblot from lysates of S2R+ cells expressing hPIP4K. (ii) S2R+ cell-free lysate expressing PIP4 kinases (10 μg) from indicated human isoforms was incubated for 16 h at 30°C with physiological (6 μM) concentration of PI5P and 0.5 μCi [γ^32^P] ATP. The assay reaction was terminated by adding 2.4 M HCl followed by methanol and chloroform. The lipids were extracted and resolved on TLC plate using chloroform:methanol:water:25% ammonia (45:35:8:2). UTC, untransfected cells; hPIP4K2A^WT^, human PIP4 kinase α isoform wild-type; hPIP4K*2C*^WT^, human PIP4Kinase γ isoform wild-type; hPIP4K*2*C ^KD^. (**B**) Graph representing average cell size measurement (μm^3^) as mean ± S.E.M. for heterologous reconstitution of *hPIP4K2C* as compared with *AB1Gal4/+;dPIP4K^29^, n*=7 or more, **P-value <0.05. (**C**) Immunoblot showing the comparison of expression between of *hPIP4K2C^WT^* or *hPIP4K2C^KD^* from salivary gland lysates and probed with anti-GFP. Tubulin was used as the loading control. (**D**) Confocal images of salivary glands expressing *hPIP4K2C*^WT^ or *hPIP4K2C*^KD^ where green represents GFP-tagged transgenic protein expressed in *dPIP4K^29^* and DAPI stains nucleus shown in blue. Scale bar indicated at 20 μm. Images are contrast adjusted for representation. (**E**) Graph representing average cell size measurement (μm^3^) as mean ± S.E.M. for heterologous reconstitution of *hPIP4K2C^WT^* or *hPIP4K2C ^KD^* as compared with *AB1Gal4/+;dPIP4K^29^, n*=7 or more, *P-value <0.01, **<0.05.

We tested the ability of *hPIP4K2C* that shows very low specific activity *in vitro* to rescue cell size. *hPIP4K2C* was expressed in *Drosophila* S2R+ cells (Figure 4A,i) and using a cell lysate we measured its enzymatic activity in comparison with hPIP4K2A. As previously reported, we found that while hPIP4K2A showed high specific activity; under these conditions, no activity was detected for hPIP4K2C (Figure 4A,ii). When expressed in *dPIP4K^29^* salivary glands (*AB1> hPIP4K2C^WT^*; *dPIP4K^29^*), *hPIP4K2C* was able to rescue the reduced cell size (Figure 4B). To test the requirement of *hPIP4K2C* activity in the rescue of cell size, we generated a mutant version that is not able to bind and hydrolyse ATP (*hPIP4K2C^KD^*) [14]; as expected, in an *in vitro* assay, this mutant enzyme also showed no detectable activity (Figure 4A,ii). When *hPIP4K2C^KD^* (*AB1> hPIP4K2C^KD^*; *dPIP4K^29^*) was expressed in salivary glands, its protein levels and localisation were similar to that of *hPIP4K2C* (Figure 4C,D). Surprisingly, we found that *hPIP4K2C^KD^* was unable to rescue the reduced cell size in *dPIP4K^29^* (Figure 4E). This observation suggests that the enzymatic activity of *hPIP4K2C* is required for its ability to support cell size *in vivo*.

## Discussion

Although a number of recent studies have uncovered key physiological roles for PIP4K in multiple model systems, their relationship with the enzymatic activity of these proteins remains unresolved. On the one hand, mammalian genomes typically contain three genes which produce polypeptides with more than 10000-fold range PIP4K activity *in vitro* between them. On the other hand, invertebrate genomes typically contain a single PIP4K gene. Remarkably, while the *C. elegans* enzyme appears to have very low specific activity *in vitro*, recapitulating that seen for *hPIP4K2C*, the *Drosophila* enzyme appears to have high specific activity similar to *hPIP4K2A.* To understand the relationship between *in vitro* activity and cellular function, we reconstituted a protein null mutant of *Drosophila* PIP4K (*dPIP4K^29^*) individually with each of the mammalian enzymes. Since the *Drosophila* genome contains only a single gene encoding PIP4K, this setting offers an experimental model where any human PIP4K isoform can be selectively reconstituted in PIP4K null cells to study the requirement of enzymatic activity for *in vivo* function. The three mammalian PIP4K isoforms have been reported to heterodimerise with one another [21,22]; however in our experimental model, there is no possibility of heterodimerisation between multiple isoforms and phenotypic outcomes will reflect the activity of only the polypeptide that is being reconstituted. We found that the ability of PIP4K to rescue cell size in *dPIP4K^29^* was dependent on its kinase activity. Thus the rescue of cell size offers a phenotype in the context of which the requirement of enzymatic activity *in vivo* can be studied.

Remarkably, we found that each of the three human isoforms of PIP4K (*hPIP4K2A, hPIP4K2B* and *hPIP4K2C*) was able to increase the salivary gland cell size when individually reconstituted in *dPIP4K^29^*. Importantly, when expressed at equivalent protein levels, the ability of each of the human isoforms to increase cell size when reconstituted in *dPIP4K^29^* was not significantly different (Figure 3F). Since these three human PIP4K isoforms have vastly differing *in vitro* activities, our results imply that *in vitro* activity is not a direct correlate of *in vivo* function in this model.

Most surprisingly, we found that *hPIP4K2C* that is reported to have minimal enzymatic activity *in vitro* was able to rescue the reduced cell size in *dPIP4K^29^* just as well as the other two isoforms (*hPIP4K2A, hPIP4K2B*) with substantially higher enzyme activity. Since this observation was made when the isoforms were individually reconstituted into *dPIP4K^29^*, it seems unlikely that this polypeptide with minimal catalytic activity mediates rescue via dimerisation with another isoform of greater specific activity. Further, we found that an ATPase dead version of *hPIP4K2C* was unable to rescue the cell size phenotype of *dPIP4K^29^* despite being expressed at equivalent protein levels to the wild-type enzyme. Thus it is likely that *hPIP4K2C* does indeed have catalytic activity *in vivo* but that under the conditions of the *in vitro* assay used, this enzyme shows very low specific activity. The nature of the missing factors that are available *in vivo* but missing *in vitro* remain unknown. However a number of scenarios are possible including accessory proteins, small molecule cofactors or the lipid environment of the vesicular compartment to which the enzyme has been reported to localise in mammalian cells. Interestingly, a recent paper has reported the development of a kinase inhibitor specific to *hPIP4K2C* that is able to ameliorate the effects of mutant Huntingtin protein expressed in cultured mammalian cells [7]. Treatment with this inhibitor also resulted in elevation in the levels of PI5P, PI3P and PI(3,5)P_2_; presumably these results are a reflection of ongoing *hPIP4K2C* activity in cells. Previous measurements of the intrinsic ATPase activity of the human isoforms have shown that *hPIP4K2C* is only two- to three-fold less active than *hPIP4K2A, hPIP4K2B* [14]. Thus, it is possible that the low specific activity observed *in vitro* for this enzyme reflects the requirement for an accessory factor required to facilitate PI5P binding to the enzyme. Interestingly, although the *dPIP4K* gene only encodes a single isoform, recent papers have defined phenotypes that do not require the kinase activity of the enzyme [11] *in vivo* and in this study we defined cell size regulation as a phenotype that requires the kinase activity of the enzyme. The mechanism by which the kinase-independent actions of dPIP4K are mediated remains unclear and will be a subject of future analysis. However the presence of kinase-independent effects of dPIP4K *in vivo* presumably reflect the presence of cellular mechanism to inhibit the wild-type activity of the dPIP4K polypeptide that is normally very high and comparable with that of *hPIP4K2A*. The discovery of such mechanisms may inform the strategy by which the activity of the human isoforms is controlled in cells and suggest novel mechanism for tuning PIP4K function for therapeutic applications.

## Supporting information

**Supplementary Figure:** 

## Abbreviations

dPIP4K: *Drosophila* PIP4K
mTOR: *m*ammalian *T*arget *o*f *R*apamycin
PIP4K: phosphatidylinositol 5 phosphate 4-kinase
PIP5K: phosphatidylinositol 4 phosphate 5-kinase
PI4P: phosphatidylinositol 4-phosphate
PI5P: phosphatidylinositol 5-phosphate
PI(4,5)P2: phosphatidylinositol 4,5 bisphosphate
