# Supplementary figures and images for: Functional analysis of the biochemical activity of mammalian phosphatidylinositol 5 phosphate 4-kinase enzymes
Source: Biosci Rep. 2019 Feb 19;39(2):BSR20182210. doi: 10.1042/BSR20182210 (PMC6379509; doi:10.1042/BSR20182210)

A.

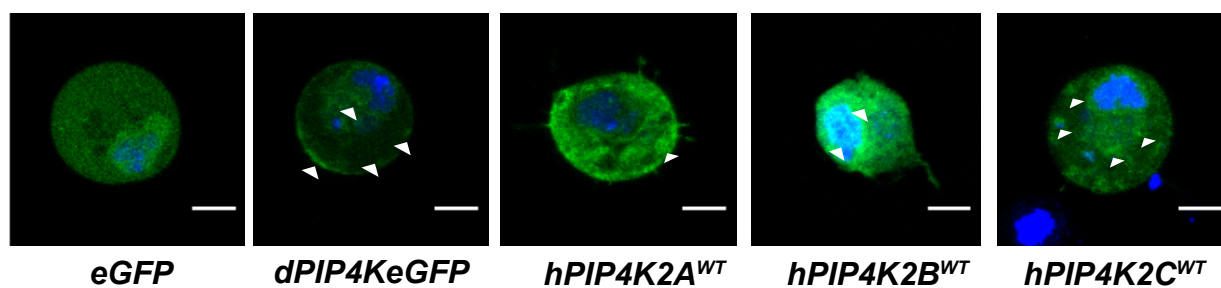

B. i.

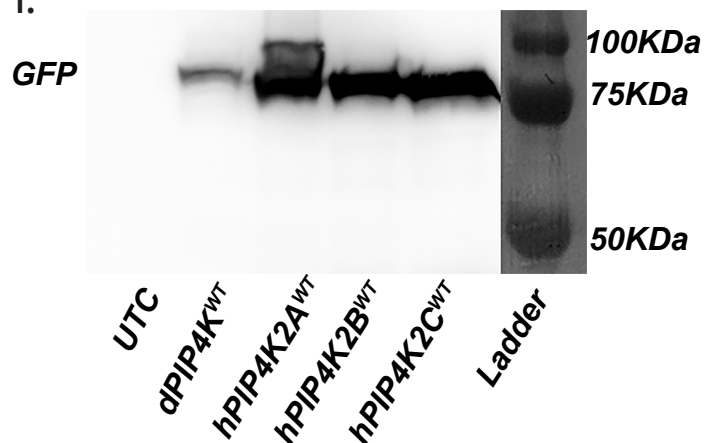

ii.

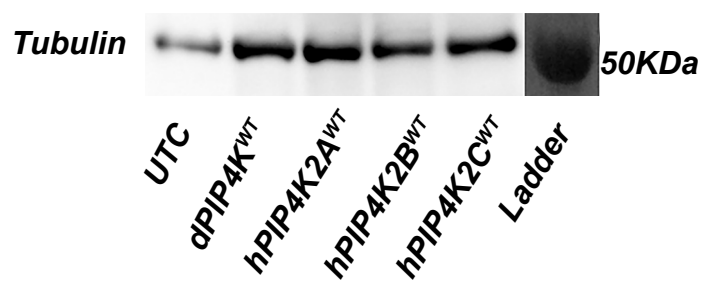

C.

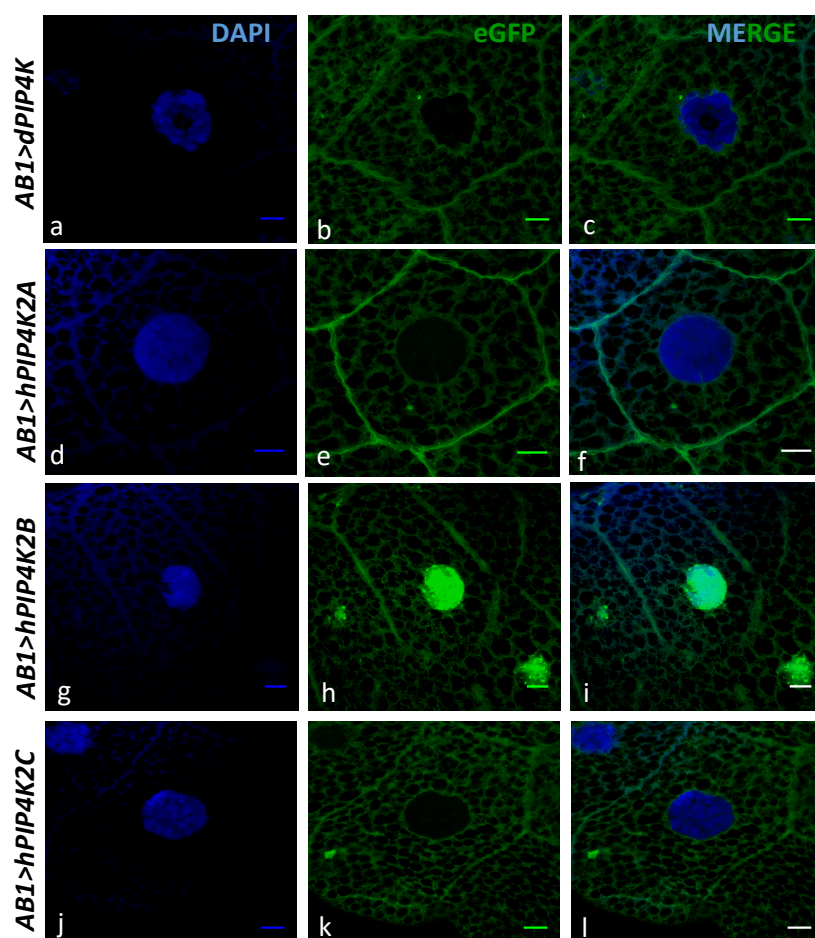

Supplement: Supplementary file 1 [file bsr-39-bsr20182210_Supp1.pdf]
